# Prevalence of *Cryptosporidium* spp. in Camels and Involved People in Yazd Province, Iran

**Published:** 2012

**Authors:** A Sazmand, A Rasooli, M Nouri, H Hamidinejat, S Hekmatimoghaddam

**Affiliations:** 1 Institute of Applied-Scientific Education of Jihad-e-Agriculture, MolaSadra Center, University of Aplied Science and Technology, Yazd, Iran; 2 School of Veterinary Medicine, Shahid Chamran University of Ahvaz, Ahvaz, Iran; 3 Department of Laboratory Sciences, School of Paramedicine, Shahid Sadoughi University of Medical Sciences, Yazd, Iran

**Keywords:** *Cryptosporidium*, Camel, Zoonotic, Public Health, Iran

## Abstract

**Background:**

Although infection of dromedary camels with *Cryptosporidium* spp. is rare in Iran, it is considered a zoonotic threat to the keepers and herders of camels. Thus we investigated the prevalence of *Cryptosporidium* in these two hosts in Yazd Province, a semi-arid region in center of Iran.

**Methods:**

This study was conducted during 4 seasons (winter 2008, summer 2009, winter 2009 and summer 2010). Fecal samples (n=200) were collected from live camels. Also, 100 abomasal mucosa and related fecal samples of the slaughtered camels were investigated. Stool samples from 100 individuals who were in persistent contact with camels were also obtained. After staining by modified Ziehl-Neelsen method, the prepared specimens were studied microscopically. Results were analyzed using SPSS 16.

**Results:**

The rate of infection in feces and abomasal mucosa of camels were 20.33% and 12%, respectively. In addition, simultaneous fecal and mucosal infection was detected in 3 cases in winter. Statistical analyses showed no significant relation between infection and age of camels, as well as their sex and the season. Cryptosporidiosis in people who were in long-term contact with camels was also investigated microscopically by obtaining stool samples of 100 individuals (50 in summers, 50 in winters), 24 of them being infected with *Cryptosporidium* spp. The rate of infection was higher in winter than summer (16/50 compared with 8/50).

**Conclusion:**

The prevalence of *Cryptosporidium* spp. in camels and involved humans in Yazd Province is relatively considerable and of public health importance.

## Introduction

Cryptosporidiosis is one of the important zoonotic diseases caused by *Cryptosporidium* spp. and its route of transmission is fecal-oral. Many vertebrates, including human beings, are affected by pathologic changes induced by this parasite (1). This pathogenic protozoan causes chronic diarrhea in those who have immune-suppression, but may induce only acute self-limiting enteritis in those with intact immune system (2). Cryptosporidiosis in man was first reported in a patient suffering from acute enterocolitis (3). About 2% of the examined stools were positive for this parasite in the US people (4). The incidence of cryptosporidiosis in people ranges from 1% to 10%, and its dependence on geography, standard of hygiene, season, age, proximity to farms and persistent contact with animals is well-known (5). Infection with this parasite is a serious problem because of lack of widespread access to efficient therapy. Besides its medical importance, infection in animals may cause enormous economic losses because of high infection rate and decreased productivity as a result of emaciation and general malaise in diseased animals (6).

One-humped camel (*Camelus dromedarius*) is an important multipurpose animal of arid and semi-arid parts of the world, including Iran. According to the last enumeration in 2010, there have been about 154000 camels in Iran, 21830 of them counted in the Yazd Province (7). According to researchers’ works, *C. muris*, *C. parvum* and *C. andersoni* are 3 species found in camelids (8–10). The rate of fecal infection of camels to a *Cryptosporidium muris*-like parasite was 3.25% (11). Iranian researchers indicated this infection in camels from different regions (12–14), but no positive cases were found in 110 camels aging 3-8 months in Tunisia (15). Cryptosporidiosis in human and animals has been studied in various parts of the country. Nouri et al. (16, 17) reported the incidence of asymptomatic cryptosporidiosis in sheep and cattle and the people dealing with them to be 13% and 1.7%, respectively. The aim of this study was to determine the infection rate in asymptomatic camels and also individuals in contact with them in the Yazd Province, Iran.

## Materials and Methods

### Study area

The study was carried out in the Yazd Province; an arid region in center of Iran located 677 km south east of Tehran. Being located near the central mountains, far from the sea, adjacent to the deserts and in the shadow rainy region, Yazd has a climate which mostly resembles dry desert climate with the mean temperatures of 30.67 and 8.36 °C in summer and winter, respectively. Little rain along with high water evaporation, relatively low dampness, heat and great temperature changes are among the factors making this province one of the driest parts of Iran.

### Sampling and investigation

In four seasons (winter 2008, summer 2009, winter 2009, summer 2010), totally 200 fecal samples (50 in each season) were collected from live asymptomatic camels. In addition, 100 abomasal mucosa (50 in summers, 50 in winters) and 100 related fecal samples (50 in summers, 50 in winters) of slaughtered asymptomatic camels were investigated. Stool samples from 100 healthy individuals (50 in summers, 50 in winters) who were in persistent contact with camels (including farmers, slaughterhouse workers and veterinary students) were also obtained and concentrated by formalin-ethyl acetate method (18). Prepared slides were stained using modified Ziehl-Neelsen method (19). Microscopic examination was performed by two experienced parasitologists for the presence of *cryptosporidium* oocysts, which were red round shape oocysts in green background with four sporozoites in 100x magnification.

### Statistical analysis

The age and sex of camels and season were recorded, and the camels were divided into three groups (<5, 5-10, and >10 years old). Data were handled using SPSS 16. Chi-Square test was used to compare different independent variables. *P*-value <0.05 was considered as statistically significant.

## Results

From the 300 fecal specimens, 61 (20.33%) were positive for *Cryptosporidium*. Of 100 abomasal mucosa specimens, 12 (12%) were also positive, including 3 camels which showed simultaneous fecal and abomasal infection in summer. There was no significant correlation between the infection and age, sex or season (*P*>0.05). Microscopic investigation of the stool of above-mentioned 100 individuals showed *Cryptosporidium* organisms in 24 of them, more in winter than summer (16 out of 50 compared with 8 out of 50) (Table 1).

**Table 1:** The infection rate of *Cryptosporidium* spp. in camels and involved people

Samples	Season		Age group (Camels)			Sex (Camels)	
	Summer	Winter	5 ≥	5 < ≥ 10	> 10	Male	Female
	
	% (No.)		% (No.)			% (No.)					
Camels feces	18 (27/150)	22.67 (34/150)	19.2 (24/125)	20.72 (29/140)	22.86 (8/35)	19.82 (45/227)	21.92 (16/73)
Abomasal mucosa	12 (6/50)	12 (6/50)	15.62 (5/32)	5.66 (3/53)	26.67 (4/15)	13.23 (9/68)	9.37 (3/32)
Human feces	16 (8/50)	32 (16/50)	-	-	-	-	-

## Discussion

Results of the present study showed *Cryptosporidium* infection in 24% of camel-involved individuals. Since most of the sampled people in the present study were poor foreigner immigrants living in the study area, the infection rate was high likely due to their low hygiene conditions. To our knowledge, *C. muris*, *C. parvum* and *C. andersoni* are 3 species that infect camelids (8–10). Three independent studies performed in different parts of Iran showed the prevalence of *Cryptosporidium* isolated from camels to be 37.9%, 16.9%, and 1.9%, respectively (12–14), but no positive cases were found in Tunisian camels (15). The present study shows no significant difference in infection rate in two climatic situations; cold-dry and hot-dry. Many scientists have studied effects of season on occurrence and prevalence of the disease, and have reached different results. According to Garber et al. and Mohammed et al., the prevalence of cryptosporidiosis in calves and cattle was higher in winter (2, 20). However, in the study by Becher et al., season had no effect on prevalence of the infection (21). These contradictions may be due to climatic differences in the study areas or husbandry management systems. The Yazd Province has two climates in the year; cold-dry and hot-dry, and cannot be regarded wet at all. Therefore, comparing our findings on the effect of season on cryptosporidial infection rate with that of previous studies is very difficult.

In the present study, age of animals had no significant correlation with prevalence of infection. In this regard, two things must be segregated, disease and asymptomatic infection. Most of the previous studies were done on animal neonates with clinical signs of infection, but asymptomatic carriers are especially important regarding dissemination of infection. Our study indicates that camels of every age may affect public health. On the other hand, camels in the study area move freely in deserts almost all the year, and are gathered for only a short period. Researches on the effect of age on prevalence of cryptosporidiosis in wild animal populations showed that infection rate did not depend on age of animals (22, 23); this is similar to what happens to camels raised in the Yazd Province. In this study there was no significant difference between infection rates of male and female camels. Razavi et al., also could not find a relationship between sex of camels and infection rate (12). In their report, age of camels also had no effect on prevalence of cryptosporidiosis. Our examined camels had no typical signs of the disease at the time of sampling, and as the molecular study by Keshavarz et al. suggested the zoonotic mode of transmission as the main route of spread of *Cryptosporidium* in Iran (24), camels may be assumed as asymptomatic carriers of *Cryptosporidium* to human and other animals in the Yazd Province.
